# Testing for the Endogenous Nature between Women's Empowerment and Antenatal Health Care Utilization: Evidence from a Cross-Sectional Study in Egypt

**DOI:** 10.1155/2014/403402

**Published:** 2014-07-22

**Authors:** Hassan H. M. Zaky, Dina M. Armanious, Mohamed Ali Hussein

**Affiliations:** ^1^School of Humanities and Social Sciences, The American University in Cairo and Social Research Center of The American University in Cairo, New Cairo 11835, Egypt; ^2^Department of Statistics, Faculty of Economics and Political Science, Cairo University, Giza 12613, Egypt; ^3^Faculty of Commerce, South Valley University, Qena 83523, Egypt

## Abstract

Women's relative lack of decision-making power and their unequal access to employment, finances, education, basic health care, and other resources are considered to be the root causes of their ill-health and that of their children. The main purpose of this paper is to examine the interactive relation between women's empowerment and the use of maternal health care. Two model specifications are tested. One assumes no correlation between empowerment and antenatal care while the second specification allows for correlation. Both the univariate and the recursive bivariate probit models are tested. The data used in this study is EDHS 2008. Factor Analysis Technique is also used to construct some of the explanatory variables such as the availability and quality of health services indicators. The findings show that women's empowerment and receiving regular antenatal care are simultaneously determined and the recursive bivariate probit is a better approximation to the relationship between them. Women's empowerment has significant and positive impact on receiving regular antenatal care. The availability and quality of health services do significantly increase the likelihood of receiving regular antenatal care.

## 1. Introduction

In 2000, at the millennium summit, all 191 United Nations member states signed the millennium declaration which sets eight Millennium Development Goals (MDGs) to be achieved by the year 2015. The fifth Millennium Development Goal calls for improving maternal health care and includes a target of reducing the maternal mortality ratio by three quarters between 1990 and 2015 and achieving universal access to reproductive health through antenatal care coverage. If the world achieves the fifth Millennium Development Goal by 2015, more than two million women will survive childbirth [1]. Most maternal and child deaths are preventable and avoidable with reproductive health services.

Women's relative lack of decision-making power and their unequal access to employment, finances, education, basic health care, and other resources are considered to be the root causes of their ill-health and that of their children [2]. Empowering women is critical to advancing human development and achieving progress towards the MDGs.

The International Conference on Population and Development (ICPD 1994) in Cairo emphasized that women should be empowered to practice control over their health and reproductive rights. Additionally, this conference stated that the level of antenatal and postnatal care is affected by the availability of health services and the demand by pregnant women and mothers for these services [3]. The Women Deliver Conference [4], held in London in October 2007, demonstrated that maternal and newborn health is a key factor to the economic growth and the social fabric of the developing nations. It emphasized that gender equality and women's empowerment are critical for development efforts to reduce poverty, improve health, and achieve economic stability and growth.

Traditionally, women's empowerment and the use of antenatal health care are analyzed separately. Studies identified socioeconomic and demographic determinants of antenatal health care utilization among women in Egypt [5, 6]. Other studies discussed the determinants of women's empowerment in Egypt [7, 8]. Some other studies examined the impact of women's empowerment on antenatal health care utilization [9–11]. Our review of these studies confirmed that the simultaneous relationship between empowerment and antenatal health care has not been studied in Egypt. To fill this gap, the study aims to model and identify the interactive relationship between women's empowerment and the use of antenatal health care. The study explores if women's empowerment and antenatal health care utilization are dependently determined or not. Both the univariate probit and the recursive bivariate probit models are utilized to test this hypothesis.

## 2. Data and Methods

### 2.1. Data

The study depends on data derived from Egypt Demographic and Health Survey [12]. This survey was conducted on behalf of the Ministry of Health (MoH), by El Zanaty and associates. The EDHS 2008 covered a representative sample of 16,527 ever married women in the age group of 15–49 years. Only married women aged 15–49 whose last birth was during the five-year period before the survey are included in the analysis because data about antenatal care was collected from this subsample only. They amounted to 8036 women.

Table 1 presents the distribution of married women whose last birth was in the five-year period before the survey according to some of their background characteristics. The majority of women (62 percent) are living in rural areas. Looking at the age distribution in Table 1, around three-fifths of those women are under the age of 30. There are few women who are aged 40 and over.

Regarding the educational level of women and their husbands, one-quartile of women never attended school, while 16 percent of husbands have never attended school. Moreover, more than half of women (52 percent) and 56 percent of husbands have completed at least the secondary level. Data of Table 1 shows that 12 percent of ever married women were working for cash at the time of the survey. Women were fairly evenly distributed across the wealth quintiles.

### 2.2. Methods

#### 2.2.1. Model Specification

Two model specifications are tested in the study. The first assumes that there is no correlation between women's empowerment and use of regular antenatal care and that women's empowerment is exogenously determined. This approach uses the univariate probit model. The second assumes that the two outcome variables are dependently determined and that the relationship is recursive. The recursive bivariate probit model is tested.

The probit model is used because both variables, receiving regular antenatal care and women's empowerment, are binary. For antenatal care, it takes the value 1 if the number of antenatal care visits is at least 4 and takes the value 0 if the number of visits is less than 4. For empowerment, this study constructs a composite indicator to express women's empowerment. Previous literature shows that creation of women's empowerment indicators are implemented by the sums of binary input variables. Women are scored one for answers to each variable that contributed to a higher degree of empowerment; otherwise they are scored zero [7–9]. However, there are other researchers who used Factor Analysis Technique to create women's empowerment indicators [13, 14]. This study will depend on the first method to create women's empowerment indicator.

The variables used to construct women's empowerment indicator are the participation of women in making decision about major household purchases, namely, daily household purchases, her health care, visiting family or relatives, how her husband's earnings and her money are used, and the use of contraception. In addition, there are some other variables which represent whether or not it is a big problem for women to go to the doctor alone or even to get a permission to go to the doctor. Internal consistency among all these variables was moderate (Cronbach's Alpha = 0.59).

An index is created using the sums of the nine binary variables to construct the indicator of women's empowerment (equal weights are given to the nine variables used to construct the indicator of women's empowerment). It ranged from 0 to 9 according to the responses of the respondents on the nine questions. The range of the responses is classified equally to two groups. According to this classification, woman is not empowered if the value of the index is from 0 to 4 and she is empowered if the index value is from 5 to 9. Categories from 0 to 4 are merged to be “0” and this means that woman is not empowered, and categories from 5 to 9 are merged to be “1” and this means that woman is empowered.

The explanatory variables are the place of residence, region, work status, age, wealth index, women's education, exposure to media (newspaper, TV, and radio), husband's education, and availability and quality of health services. Description of these variables is shown in Table 1 except for the availability and quality of health services. These two variables need special treatment as they are not readily available in the data used. The study constructs composite indicators to express them. These composite indicators are created by Exploratory Factor Analysis technique (EFA). There are several methods for obtaining factor extraction and rotation [15]. This study uses principal component as an extraction method and VARIMAX as a rotation method.

In 2008 EDHS, women were asked to mention whether or not there is a problem on some issues related to the availability of health services. These issues are about distance to the health facility, having to take transportation, and availability of female health provider. The indicator of availability of health services is constructed using these variables and by applying the Factor Analysis Technique. One factor is extracted and this factor explains about 59 percent of the total variations among the data and it represents the availability of health services. Internal consistency among these variables was moderate (Cronbach's Alpha = 0.56).

Similarly, women who reported that they received antenatal care, tetanus toxoid injections, or other medical care unrelated to the pregnancy are asked whether they were weighed, their blood pressure measured, and urine and blood samples taken during any of the visits they made to a medical provider during their pregnancy. Those women are also asked whether they had been told about the signs of pregnancy complications, and, if they were told, whether they received any information about where to go if they experienced any complications. Finally, women are also asked whether they were given or had bought iron tablets or syrup [12]. Using these variables, Factor Analysis Technique is also used to construct the indicators of quality of health services. Two factors are extracted and they explain about 65 percent of the total variations among the data.  Table 2 shows the rotated factor loadings for these factors. Internal consistency among all these variables was high (Cronbach's Alpha = 0.74).

The results of the factor analysis show that the first factor includes the following four variables: blood and urine samples were taken, blood pressure was measured, and woman was weighted. This factor explains about 47 percent of the variation and it represents “content of antenatal care.” The second factor includes the following three variables: woman was told about the signs of pregnancy complications, was told where to go if she had any of these complications, and was given or bought any iron tablets or syrup. This factor explains about 18 percent of the variation and it represents “treatment with complications.”

Our second specification relies on the bivariate probit model which is a joint model for two binary outcomes that are allowed to be correlated with each other. Greene [16] shows that estimators from the bivariate probit model become consistent and efficient when the dependent variables in the two-equation model are binary, and omitted variables are correlated with each other. Maddala (1983) listed the bivariate probit model with endogenous dummy model among the recursive models. The recursive structure builds on a first reduced form equation for the potentially endogenous dummy and a second structural form equation determining the outcome of interest:

(1) $$\begin{array}{c} Y_{1i}^{*}={\tilde{\beta }}_{1}X_{1i}+u_{1i}\\ Y_{2i}^{*}={\tilde{\beta }}_{2}X_{2i}+u_{2i}=\delta_{1}Y_{1i}+{\tilde{\delta }}_{2}Z_{2i}+u_{2i}, \end{array}$$

where *Y*_1i_* and *Y*_2i_* are latent variables, and *Y*_1*i*_ and *Y*_2*i*_ are binary variables following the rule

(2) $$\begin{array}{c} Y_{Li}=\{\begin{array}{cc} 1 & \text{if}\;\;Y^{*}>0\\ 0 & \text{if}\;\;Y^{*}\leq 0; \end{array}\;L=1,2. \end{array}$$

*X*_1*i*_ and *Z*_2*i*_ are vectors of exogenous variables, *β*_1_ and *δ*_2_ are parameter vectors, *δ*_1_ is a scalar parameter, and ${\tilde{\beta }}_{2}=(\delta_{1}{\tilde{\delta }}_{2}\tilde{)}$. In the second specification, the error terms are assumed to be independently and identically distributed as bivariate normal [17].

In the study, *Y*_1_ refers to women's empowerment and *Y*_2_ is receiving regular antenatal health care. *Y*_1_* is a latent variable which denotes the level of women's empowerment. *Y*_2_* is a latent variable which denotes the probability that the woman will receive regular antenatal health care.

The exogeneity test in the recursive bivariate probit model arises from allowing the correlation between the error terms (*u*_1*i*_ and *u*_2*i*_) to be nonzero. The exogeneity test can be constructed from the following null and alternative hypotheses:

(3) $$\begin{array}{c} {\text{H}}_{0}:\rho =0,\;\;{\text{H}}_{\text{a}}:\rho \neq 0, \end{array}$$

where *ρ* is the coefficient of correlation between the residuals from (1). H_0_ corresponds to the assumption of exogeneity of women's empowerment variable in the antenatal care equation [18].

## 3. Results

The study estimates both the univariate and recursive bivariate probit models and the results are shown in Table 3. The univariate probit model assumes that women's empowerment is exogenously determined. According to the results of this model and unlike our a priori expectations, women's empowerment does not have any significant effect on receiving regular antenatal care.

Since the main objective of the study is to test the simultaneous nature between women's empowerment and receiving antenatal care, so the recursive bivariate probit model is estimated. The estimation results and the exogeneity test, shown in Table 3, provide significant evidence in favor of supporting a simultaneous relationship between women's empowerment and receiving regular antenatal care (the likelihood ratio test is significant with *P* value less than 10 percent). This confirms our a priori expectations that receiving regular antenatal care and women's empowerment are simultaneously determined and that the recursive bivariate probit model specification is a better representation of this relationship than the univariate probit model. The results of the recursive bivariate probit, shown in Table 3, demonstrate that women's empowerment increases the probability of receiving at least four antenatal care visits at less than 5% significance level.

The results also report the effects of other socioeconomic variables on receiving regular antenatal care. There is a significant relationship between both women's education and husband's education and receiving regular antenatal care. Women who have achieved secondary or higher education are more likely to receive at least four antenatal care visits compared with women who have never attended school. Moreover, women whose husbands have achieved secondary education or higher are more likely to receive regular antenatal care compared with those whose husbands have never attended school.

Table 3 indicates that wealth index significantly increases the likelihood of receiving regular antenatal care. Woman from the richest quintile is more likely to receive regular antenatal care compared with woman from the poorest quintile. Additionally, it can be noticed that women's work status has a significant impact on receiving regular antenatal care. Women who work for cash are more likely to receive at least four antenatal care visits compared with women who do not work for cash.

The findings of this study show that the availability of health services significantly increases the likelihood of receiving regular antenatal care. Additionally, the higher the level of indicators of quality of health services is, the more likely the women will receive at least four antenatal care visits.

Table 3 also provides the determinants of women's empowerment estimated by the recursive bivariate probit model. Data of Table 3 shows that women's age is positively correlated with women's empowerment. Additionally, women's education is associated with higher women's empowerment,   implying the possibility that women who have achieved secondary or higher education are more empowered than those who have never attended school. Women's working status contributes to the higher level of women's empowerment, suggesting that women who work for cash are more empowered than those who do not work for cash.

**Figure 1:**
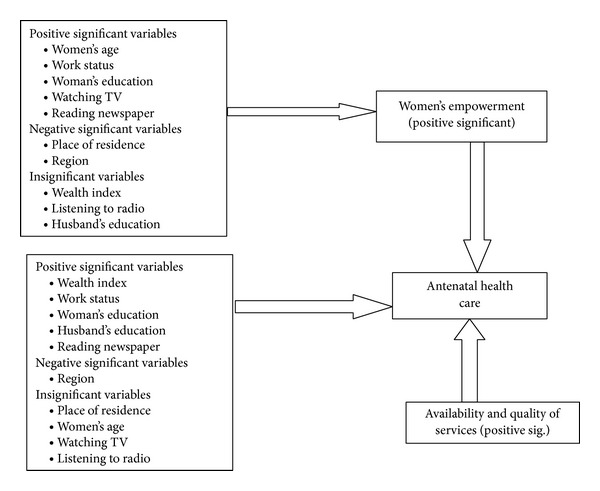
Summary results of the recursive bivariate model.

The results indicate that exposure to the media significantly increases the likelihood of women's empowerment. Table 3 shows that women who are reading newspapers or watching television are more likely to get involved into decision-making processes, compared with women who are not reading newspapers or watching television.  Figure 1 summarizes the basic results of the recursive bivariate model.

## 4. Discussion

The findings reveal that receiving regular antenatal care and women's empowerment are simultaneously determined and therefore the recursive bivariate probit model specification is more appropriate than the two separate univariate probit models. This result is in agreement with the findings from Tajikistan [19]. Additionally, the study shows that women's empowerment increases the probability that the woman receives at least four antenatal care visits.

The findings of this study show that wealth index significantly increases the likelihood of receiving regular antenatal care. This result is consistent with evidence from Ahmed [6] and Bloom et al. [9], who argued that women who are wealthy were more likely to attend antenatal care than those from poorer households.

The results show that women's education is correlated with receiving regular antenatal care. As from literature in Tajikistan, it is proved that women who have achieved secondary or higher education are more likely to receive at least four antenatal care visits compared with women who have never attended school [19].

The study shows that the higher the level of indicators of quality of health services, the more likely the women to receive at least four antenatal care visits. This confirms a previous study by Zaky et al. [11] which showed that quality of health services has a positive impact on the number of antenatal care visits.

Similar to the results by Kamiya [19] in Tajikstan, one of the major findings is that women's working status is significantly (*P* value less than 1 percent) correlated with women's empowerment.

With regard to education, this study confirmed the findings of the study by Kamal [8] in Egypt showing that women's education plays an important role in raising the level of women's empowerment. Also, this study and another study by Shafei [7] in Egypt indicate that exposure to the media significantly increases the likelihood of women's empowerment.

### 4.1. Limitations of the Study

Because of data limitations, where women' empowerment module is limited to some questions, the analysis of future women empowerment studies could benefit from collecting additional data about women's role and activities such as participation in political life, violence at home, gender preference, getting a job and finance, and measuring their self-confidence to better capture all dimensions of women's empowerment. Also, this study lacked some variables related to availability and quality of health services, which are often considered key factors in the ability to seek services, such as number of opening hours for the facilities, waiting times at the health facility, providers' skills in counselling, and health education skills. Despite these limitations, this study tested for the first time the interactive relation between women's empowerment and the use of antenatal health care in Egypt using a recursive model. Accordingly, it provides important policy implications on the role of women's empowerment and its impact on women's health. In addition, the study uses the most recent available data based on a representative sample of ever married women aged 15–49 years in Egypt. Accordingly, the results are generalizable and are not limited.

## 5. Concluding Remarks and Recommendations

The study attempted to examine the interactive relation between women's empowerment and the use of maternal health care and whether women's empowerment and receiving regular antenatal care are simultaneously determined. The recursive bivariate probit model is used to achieve this goal. The findings of the model show that women's empowerment and receiving regular antenatal care are simultaneously determined. Furthermore, the findings confirm that women's empowerment is crucial in improving maternal health care in the developing countries.

Based on the main findings of the study, the following recommendations can be introduced.

- More efforts are needed to reduce inequity among Egyptian women.
- More governmental efforts are suggested to target poor women and increase their awareness of the risks they might be exposed in order to encourage them to utilize antenatal health care services.
- Awareness programs about antenatal care are advisable to target less educated women and inform them about the advantages of antenatal care utilization.
- Collecting information about how women who had antenatal care are satisfied with the services offered to them and the problems they face during receiving the antenatal care.
- Collecting information about the complaints of antenatal health care services is important to solve these problems and improve the quality of the services.
- Further research is needed, using more updated measures of women's empowerment, on the relationship between women's empowerment and antenatal health care utilization, which are advised to draw more robust empirical results.

## Figures and Tables

**Table 1:** Distribution of married women whose last birth was during the five-year period preceding the survey according to women's background characteristics, 2008.

Items	Percentage	Number
Place of residence		
Urban Rural	38.2 61.8	3020 5016
Region		
Urban governorates Urban lower Egypt Rural lower Egypt Urban upper Egypt Rural upper Egypt Frontier governorates	16.4 10 34.3 10.8 27.1 1.4	1075 712 2282 945 2564 458
Current age 15–19	3.5	276
20–24 25–29 30–34 35–39 40–44 45–49	24.4 33.7 20.9 11.7 4.9 0.9	1943 2631 1724 963 416 83
Educational attainment		
No education Incomplete primary Complete primary Incomplete secondary Complete secondary Higher	25.3 6.7 3.5 12.2 39.3 13	2186 553 268 983 3073 973
Work status		
Not working for cash Working for cash	88.4 11.6	7100 936
Reading newspaper		
No Yes	76.2 23.8	6168 1868
Watching TV		
No Yes	2.8 97.2	284 7752
Listening to radio		
No Yes	40.1 59.9	3453 4583
Wealth index		
Poorest Poorer Middle Richer Richest	19.3 19.7 21 20.6 19.4	1767 1641 1669 1515 1444
Partner's educational attainment		
No education Incomplete primary Complete primary Incomplete secondary Complete secondary Higher	16 10.6 5.1 12.5 39.5 16.3	1363 868 387 976 3223 1219

Total	100	8036

**Table 2:** Rotated factor loadings of quality of health services.

Variables	Factors	
	First	Second
Blood pressure measured	**0.782**	0.099
Blood sample taken	**0.799**	0.186
Urine sample taken	**0.801**	0.176
Weight	**0.782**	0.071
Told about the signs of pregnancy complications	0.158	**0.776**
Given any iron tablets or syrup	0.099	**0.804**

**Table 3:** Univariate and recursive bivariate probit model estimates: results of the two specifications.

Variables	Univariate probit		Recursive bivariate probit			
	Regular ANC		Regular ANC		Women's empowerment	
	Coef.	St. error	Coef.	St. error	Coef.	St. error
Women's empowerment	−0.024	0.041	1.166∗∗	0.343		
Place of residence						
Rural (ref.)						
Urban	−0.007	0.005	0.147	0.167	−0.282∗∗	0.135
Region						
Urban governorates (ref.)						
Urban lower Egypt Rural lower Egypt Urban upper Egypt Rural upper Egypt Frontier governorates	−0.129 −0.333∗ −0.309∗ −0.519∗ −0.175∗∗∗	0.089 0.071 0.080 0.074 0.100	−0.039 −0.388∗∗∗ −0.295∗ −0.750∗ −0.512∗	0.096 0.212 0.078 0.178 0.115	0.217∗∗ 0.375∗∗ −0.096 −0.142 −0.619∗	0.084 0.146 0.072 0.145 0.093
Wealth index						
Poorest (ref.)						
Poorer Middle Richer Richest	0.206∗ 0.327∗ 0.573∗ 0.820∗	0.051 0.056 0.066 0.082	0.173∗∗ 0.285∗ 0.540∗ 0.737∗	0.056 0.066 0.083 0.118	0.004 0.006 0.116∗∗∗ 0.095	0.051 0.056 0.066 0.078
Reading newspaper						
No (ref.)						
Yes	0.038	0.05	0.116∗∗	0.056	0.233∗	0.049
Watching TV						
No (ref.)						
Yes	0.013	0.093	0.130	0.092	0.252∗	0.092
Listening to radio						
No (ref.)						
Yes	0.014	0.036	0.022	0.033	0.039	0.036
Women's age						
15–24 (ref.)						
25–39 40–49	−0.084 0.029	0.057 0.131	−0.045 0.043	0.057 0.099	0.174∗ 0.346∗	0.037 0.079
Work status						
Not working for cash						
Working for cash	0.058	0.063	0.215∗∗	0.085	0.529∗	0.072
Women's education						
No education (ref.)						
Incomplete primary Complete primary Incomplete secondary Complete secondary Higher	0.134∗∗∗ 0.044 0.129∗∗ 0.175∗∗ 0.295∗∗	0.071 0.093 0.061 0.056 0.095	0.179∗∗ 0.059 0.151∗∗ 0.242∗ 0.385∗	0.066 0.086 0.057 0.052 0.089	0.146∗∗ 0.034 0.086 0.201∗ 0.322∗	0.070 0.090 0.059 0.055 0.094
Husband's education						
No education (ref.)						
Incomplete primary Complete primary Incomplete secondary Complete secondary Higher	0.005 0.158∗∗∗ 0.048 0.083 0.259∗∗	0.064 0.087 0.064 0.056 0.082	−0.019 0.116 0.057 0.105∗∗ 0.268∗∗	0.060 0.082 0.059 0.052 0.077	−0.064 −0.047 0.033 0.087 0.127	0.062 0.083 0.063 0.055 0.079
Availability of health services	0.038∗∗	0.017	0.029∗∗	0.015		
Content of antenatal care	0.396∗	0.016	0.337∗	0.049		
Treatment with complications	0.279∗	0.019	0.237∗	0.039		
Constant	0.645∗	0.165	0.906∗∗	0.301	0.46∗∗	0.185
Rho (*ρ*)			−0.711	0.235		
LR test			3.506∗∗∗			

Source: calculated by authors using EDHS, 2008.

∗Significant at level 1%. ∗∗Significant at level 5%. ∗∗∗Significant at level 10%.
